# The Other Fellow's Point of View

**Published:** 1893-06

**Authors:** 


### Editorial, Etc.

---

THE OTHER FELLOW'S POINT OF VIEW.—One of the most difficult things in life is to regard a situation, see an object, or consider an event from a point of view not our own. The stronger our own personality, the more decided our opinions, the more difficult becomes this generosity of mental gaze. The canny Scot, while wishing for the power to see ourselves as others see us, might wisely have wished also that we might see others as they see themselves. To have opinions based on calm thought and quiet judgment, and to hold unto them, is a very good thing indeed. To insist upon your friend, who is of a different temperament and differently circumstanced, assuming those same opinions is surely insular and narrow. Insist upon your right to hold to your own point of view for yourself, and accord to your friend or your friendly enemy the same privilege. Define in your own understanding the difference between lion-like firmness and asinine obstinacy. The one is as restful and supporting as a mild-eyed Normandy steed, the other as confusing and as annoying as a kicking zebra. Many people go about the world with their opinions always in aggressive evidence. They recognize no point of view but their own. They court opposition, and regard with glee any opportunity of an attempt to convince the other fellow, not so much that *he* is entirely wrong as that *they* are entirely right. It may be well to remember that a "point" is an extremely small space, and that your own absolute *point* of view holds accommodation but for yourself. Tolerance is a very good oil with which to soften the friction of human intercourse. If you will listen with patience to the other fellow's exposition of his views, you may gain much interest therefrom. If we would discuss more and argue less, we should go far toward seeing why our opinions are not endlessly repeated in the minds of others.

You wear your own clothes, and they fit you to your own way of thinking. You do not force them upon another man who is taller, or shorter, or broader, or heavier, and expect them to fit him. Treat your point of view as you do your clothes—have it good, well fitted, and wear it with such grace as may be; and in the name of all that is comfortable, let the other fellow do the same.—*Harper's Bazar.*

